# Blood analytes of oceanic-juvenile loggerhead sea turtles (*Caretta caretta*) from Azorean waters: reference intervals, size-relevant correlations and comparisons to neritic loggerheads from western Atlantic coastal waters

**DOI:** 10.1093/conphys/coy006

**Published:** 2018-02-16

**Authors:** Nicole I Stacy, Karen A Bjorndal, Justin R Perrault, Helen R Martins, Alan B Bolten

**Affiliations:** 1 Department of Large Animal Clinical Sciences, College of Veterinary Medicine, University of Florida, 2015 SW 16th Ave, Gainesville, FL 32610, USA; 2 Archie Carr Center for Sea Turtle Research and Department of Biology, University of Florida, PO Box 118525, Gainesville, FL 32611, USA; 3 Loggerhead Marinelife Center, 14200 U.S. Hwy 1, Juno Beach, FL 33408, USA; 4 University of the Azores, Department of Oceanography and Fisheries, PT-9901-862 Horta, Azores, Portugal

**Keywords:** Juvenile, marine turtle, oceanic-juvenile stage, packed cell volume, plasma chemistry, size relationship

## Abstract

Blood analyte reference intervals are scarce for immature life stages of the loggerhead sea turtle (*Caretta caretta*). The objectives of this study were to (1) document reference intervals of packed cell volume (PCV) and 20 plasma chemistry analytes from wild oceanic-juvenile stage loggerhead turtles from Azorean waters, (2) investigate correlations with body size (minimum straight carapace length: SCL_min_) and (3) compare plasma chemistry data to those from older, larger neritic juveniles (<80 cm SCL_min_) and adult loggerheads (≥80 cm SCL_min_) that have recruited to the West Atlantic in waters around Cape Canaveral, Florida. Twenty-eight Azorean loggerhead turtles with SCL_min_ of 17.6–60.0 cm (mean 34.9 ± 12.1 cm) were captured, sampled and immediately released. Reference intervals are reported. There were several biologically relevant correlations of blood analytes with SCL_min_: positive correlations of PCV, proteins and triglycerides with SCL_min_ indicated somatic growth, increasing diving activity and/or diet; negative correlations of tissue enzymes with SCL_min_ suggested faster growth at smaller turtle size, while negative correlations of electrolytes with SCL_min_ indicated differences in diet, environmental conditions and/or osmoregulation unique to the geographic location. Comparisons of loggerhead turtles from the Azores (i.e. oceanic) and Cape Canaveral (i.e. neritic) identified significant differences regarding diet, somatic growth, and/or environment: in Azorean turtles, albumin, triglycerides and bilirubin increased with SCL_min_, while alkaline phosphatase, lactate dehydrogenase and sodium decreased. In larger neritic Cape Canaveral turtles, aspartate aminotransferase increased with SCL_min_, while the albumin:globulin ratio, phosphorus and cholesterol decreased. These differences suggest unique physiological disparities between life stage development and migration, reflecting biological and habitat differences between the two populations. This information presents biologically important data that is applicable to stranded individual turtles and to the population level, a tool for the development of conservation strategies, and a baseline for future temporal and spatial investigations of the Azorean loggerhead sea turtle population.

## Teaser

This study reports reference intervals and correlations of blood analytes of oceanic-juvenile loggerhead sea turtles from Azorean waters with body size and comparison of data to neritic loggerheads from the US Atlantic coast. The observed findings highlight conservation-relevant biological (e.g. diet, somatic growth) and habitat differences among both populations.

## Introduction

The loggerhead sea turtle (*Caretta caretta*) distinct population segments are listed as threatened or endangered under the US Endangered Species Act (NMFS and USFWS 2011). Blood provides a sample matrix for a number of different analyses, such as basic hematology and chemistry, as a diagnostic screening tool in any species during health and disease. Therefore, blood analysis has been widely used in health assessment studies and mortality investigations and is increasingly applied as an important part of the development of conservation strategies for sea turtle populations worldwide. Various studies document blood analyte data of loggerhead turtles at various life stages, including nesting or adult foraging, and from different geographic regions (Bolten *et al.*, 1992, 1994; Casal *et al.*, 2009; Deem *et al.*, 2009; Flint *et al.*, 2010; Kelly *et al.*, 2015). However, published data of the wild oceanic-juvenile life stage is limited (Delgado *et al.*, 2011).

Loggerhead turtles have complex life histories that can span ocean basins and take greater than 30 years to reach sexual maturity (Bolten, 2003a). In the North Atlantic, loggerheads hatch on beaches in the West Atlantic (primarily in the SE US), enter the sea, and most are passively carried by currents, along with some active orientation and swimming, to the region around the Mid-Atlantic Ridge; this oceanic-juvenile population spends the next decade in the open ocean in the mid-Atlantic waters around the Azores (Bjorndal *et al.*, 2000b, 2003b; Avens *et al.*, 2013; Bolten, 2003a). The connectivity of the Azorean developmental population with the nesting population in the southeastern USA has been documented using genetic markers (Bolten *et al.*, 1998).

Oceanic-juvenile loggerheads primarily consume pelagic and epipelagic gelatinous coelenterates that are caught near the surface along convergent zones and drift lines in these deep oceanic waters (Bjorndal, 1997; Frick *et al.*, 2009; Jones and Seminoff, 2013). When the turtles recruit to shallow coastal, neritic waters of the West Atlantic at ~10 years of age, they switch their diet from gelatinous prey to a more mixed diet consisting of hard shelled, benthic invertebrates (Bjorndal, 1997).

The region of the mid and Eastern Atlantic appears to have major biological importance in the development of loggerhead turtles. With reference to the geographic distance of the Azores and the Madeira Archipelagos that are ~1000 km apart, Bolten *et al.* (1993) document that loggerheads from the Madeira Archipelago are comparatively larger and thus further along in their development compared to Azorean turtles. Given these differences in life stage/development, diet and habitat, biochemical differences are expected to be reflected in plasma analytes. However, Delgado *et al.* (2011) is the only study that reports blood biochemistry data from wild oceanic-juvenile loggerhead turtles from waters around Madeira, while no reports from Azorean loggerheads exist to date.

The objectives of this study were to (1) document reference intervals of packed cell volume (PCV) and 20 plasma chemistry analytes from wild oceanic juvenile-stage loggerhead turtles from Azorean waters, (2) investigate correlations with body size (minimum straight carapace length: SCL_min_), and (3) compare plasma chemistry data to those from older and larger neritic juveniles (<80 cm SCL_min_) and adult loggerheads (≥80 cm SCL_min_) that have recruited to the West Atlantic in Cape Canaveral.

## Materials and methods

Loggerhead turtles were captured by hand or dip net during the month of November 1990 in Azorean waters. All turtles were visually examined and 3–5 ml of blood were collected within 2 min of capture from the dorsal post-occipital sinus using lithium-heparin vacutainers (Becton-Dickinson Diagnostics, Pre-Analytical Systems, Franklin Lakes, NJ, USA). After measurement of SCL_min_ (cm) and mass (kg), the animals were tagged on both front flippers and then released. For turtles with available mass, a body condition index (BCI) was calculated (Bjorndal *et al.*, 2000a).

Loggerhead turtles from Cape Canaveral, Florida, USA, were captured year-round (monthly captures during March 1992–February 1993) by trawl net (total mean trawl duration each month was 153 min for 6 trawls, resulting in a mean trawl duration of 25.5 min) as previously described (Bolten *et al.*, 1994) and samples were collected and processed as for Azorean turtles. For both locations, blood samples were immediately processed on the boat. After careful mixing of whole blood with lithium-heparin, two 75 mm capillary tubes were filled with whole blood and centrifuged for 5 min in a micro-capillary centrifuge Model MB (Damon/IEC, Needham Heights, MA, USA). PCV was recorded as the mean percent PCV of both capillary tubes. The remaining whole blood was centrifuged for 5 min at 2000× *g* (IEC Clinical Centrifuge, Needham, MA, USA), plasma was harvested, and plasma color reported. Plasma was then immediately stored in liquid nitrogen and transported to the University of Florida, Department of Biology (Gainesville, FL, USA). Samples from the Azores were shipped back to Florida in a liquid nitrogen dry shipper. Samples were stored in an ultracold freezer at –70°C until analyzed within 4 weeks of return to the University of Florida. Plasma samples were analyzed by a commercial laboratory (SmithKline Beecham Clinical Laboratories, Tampa, FL, USA) using an Olympus AU5061 autoanalyzer for the following plasma chemistry analytes: albumin, alkaline phosphatase (ALP), alanine aminotransferase (AST), aspartate aminotransferase (ALT), blood urea nitrogen (BUN), calcium (Ca), chloride (Cl), cholesterol, creatinine, globulins, glucose, iron, lactate dehydrogenase (LDH), phosphorus (P), potassium (K), sodium (Na), total bilirubin, total protein, triglycerides and uric acid. The albumin:globulin (A:G) and Ca:P ratios were calculated.

Statistical analyses were performed using MedCalc® statistical software (version 17.6, Ostend, Belgium) and IBM SPSS Statistics 24 (SPSS, Inc., Chicago, IL, USA). Mean, standard deviation, median and range are reported for all data. Additionally, reference intervals were calculated for Azorean loggerheads using 90% reference intervals and associated 90% confidence intervals based on recommendations by Friedrichs *et al.* (2012) for sample sizes greater than or equal to 20, but less than 40. Normality was assessed using the D’Agostino-Pearson test. For data that were not normally distributed, Box Cox transformations were utilized to establish reference intervals. For analytes that could not be transformed to meet the assumptions of normality, the robust method for determining reference intervals was utilized. Any outliers were identified using the Dixon-Reed outlier test; these values were not removed, but are indicated when appropriate (Kelly *et al.*, 2015).

Relationships between SCL_min_ and the measured blood analytes were determined using least-squares linear regressions when the residuals met the assumptions of normality. If the residuals could not be normalized, Spearman correlations were used to determine if the blood analytes were related to SCL_min_. These regressions or correlations were performed for each size class of interest: Azorean loggerheads (oceanic juveniles); Cape Canaveral loggerheads <80 cm SCL_min_ (i.e. neritic juveniles; FFWCC 2016); Cape Canaveral loggerheads ≥80 cm SCL_min_ (i.e. adults; FFWCC 2016); all loggerheads (Azores + Cape Canaveral). Lastly, lines-of-best-fit between the measured blood analytes and SCL_min_ were determined from Azorean loggerheads and Cape Canaveral loggerheads <80 cm SCL_min_. This size class was chosen for comparison as FFWCC (2016) suggests that loggerheads <80 cm SCL_min_ are not of breeding size, and all of the Azores samples fell below this size class cutoff. The slopes of the lines-of-best-fit between each analyte were compared using a Student’s *t* test (e.g. the slope of the line-of-best-fit between albumin and SCL_min_ for Azorean loggerheads was compared to the slope of the line-of-best-fit between albumin and SCL_min_ for Cape Canaveral loggerheads <80 cm SCL_min_).

## Results

Twenty-eight Azorean loggerhead turtles were captured and sampled. All turtles were apparently healthy (i.e. active, alert, no external wounds). There was no hemolysis detected in any of the plasma samples. Mass, morphometric and blood analyte data are presented in Table 1 (Conventional Units), and comparative data of this and previously published data from various studies in Table 2 (Conventional Units). Supplemental Tables 1 and 2 present complementary tables in Standard International (SI) units. Because only small turtles were weighed, results in kg are biased towards the smaller sized turtles and do not truly reflect the size distribution of all the study turtles.

**Table 1:** Morphometrics, body condition index and blood analyte data for oceanic-juvenile loggerhead sea turtles from the Azores sampled in November 1990 in conventional units

Parameter	Mean ± SD	Median	Range	*N*	Lower limit (90% CI)	Upper limit (90% CI)
SCL_min_ (cm)	34.9 ± 12.1	39.4	17.6–60.0	28	NA	NA
Mass (kg)	1.5 ± 0.6	1.2	1.0–2.6	8	NA	NA
BCI	1.8 ± 0.1	1.8	1.6–2.0	8	NA	NA
Albumin (g/dl)	1.0 ± 0.2	1.1	0.7–1.3	28	0.7 (0.6–0.8)	1.3 (1.2–1.4)
Albumin:globulin ratio	0.42 ± 0.04	0.42	0.30–0.50	28	0.35 (0.32–0.37)	0.49 (0.47–0.51)
ALP (U/l)	28.5 ± 10.4	26.0	11.0–52.0	28	11.4 (5.8–17.1)	45.6 (39.9–51.2)
ALT (U/l)	3.0 ± 6.5	1.0	0–28.0	28	0 (0–0.01)^a^	15.4 (6.4–33.0)^a^
AST (U/l)	154.3 ± 44.4	147.5	94.0–287.0	28	101.6 (91.8–113.3)^a^	238.0 (198.1–294.4)^a^
BUN (mg/dl)	73.6 ± 9.0	77.5	44.0^b^–82.0	28	58.8 (0–67.6)^a^	82.6 (80.7–84.3)^a^
Calcium (mg/dl)	7.6 ± 1.2	7.3	4.7–10.9	28	5.5 (4.9–6.2)	9.6 (8.9–10.3)
Calcium:phosphorus ratio	0.84 ± 0.26	0.82	0.28–1.38	28	0.40 (0.26–0.55)	1.27 (1.22–1.41)
Chloride (mmol/l)	113.1 ± 4.9	113.0	103.0–123.0	28	105.1 (102.4–107.7)	121.1 (118.4–123.7)
Cholesterol (mg/dl)	177.5 ± 68.2	163.5	92.0–361.0	28	65.3 (28.1–102.5)	289.7 (252.6–326.9)
Creatinine (mg/dl)	0.2 ± 0.04	0.2	0.1–0.3	28	0.14 (0.12–0.16)^c^	0.26 (0.24–0.28)^c^
Globulins (g/dl)	2.4 ± 0.6	2.6	1.6–3.4	28	1.4 (1.2–1.8)^d^	3.5 (3.2–3.7)^d^
Glucose (mg/dl)	116.0 ± 19.6	114.0	88.0–174.0	28	92.4 (87.6–98.0)^a^	151.3 (134.9–174.4)^a^
Iron (μg/dl)	22.3 ± 14.9	17.0	4.0–57.0	28	0 (0–5.9)	46.8 (38.7–55.0)
LDH (U/l)	64.2 ± 26.5	60.5	19.0–138.8	28	0^a^	113.1 (93.1–136.1)^a^
PCV (%)	22.0 ± 5.0	21.0	14.0–32.0	20	14.0 (11.0–17.0)	30.0 (27.0–33.0)
Phosphorus (mg/dl)	9.8 ± 2.9	9.3	5.7–16.8	28	5.1 (3.5–6.6)	14.5 (12.9–16.1)
Potassium (mmol/l)	3.7 ± 0.2	3.7	3.2–4.1	28	3.3 (3.2–3.4)	4.1 (3.9–4.2)
Sodium (mmol/l)	155.7 ± 3.3	155.0	149.0–164.0	28	150.3 (148.5–152.1)	161.1 (159.3–162.8)
Total bilirubin (mg/dl)	0.07 ± 0.06	0.10	0–0.20	28	0 (0)	0.17 (0.14–0.20)
Total protein (g/dl)	3.5 ± 0.7	3.7	2.3–4.7	28	2.1 (1.9–2.7)^d^	4.8 (4.4–5.1)^d^
Triglycerides (mg/dl)	197.9 ± 167.0	139.5	21.0–637.0	28	29.0 (16.2–50.4)^a^	576.1 (369.9–882.1)^a^
Uric acid (mg/dl)	0.9 ± 0.4	0.8	0.5–2.5^e^	28	0.6 (0.5–0.6)^a^	1.5 (1.2–2.0)^a^

Reference intervals represent the 90% confidence interval.

^a^ALT, AST, BUN, glucose, LDH, triglycerides and uric acid reference intervals calculated using Box Cox transformations, as data were non-normal.

^b^44.0 mg/dl is an outlier; the next lowest value is 57 mg/dl.

^c^Creatinine could not be transformed to a normal distribution and robust methods of determining reference.

^d^Globulins and total protein reference intervals are reported using the robust method.

^e^2.5 mg/dl is an outlier; the next highest value is 1.3 mg/dl.

**Table 2:** Comparison of published blood analytes in loggerhead sea turtles in conventional units

Reference	This study: Azores^a^	This study: Cape Canaveral^a,b^	Casal *et al.* (2009)^c^	Delgado *et al.* (2011)^d^	Kelly *et al.* (2015)^e^	Deem *et al.* (2009)^f^
Location	Azores; oceanic	Florida USA; neritic	Cape Verde; rehab	Madeira; oceanic	North Carolina USA; neritic	Florida/Georgia USA; neritic
Date	Nov 90	Mar 92–Feb 93	Aug–Sep 04	May–Jul 06	May–Nov 04–07	May–Sep 00–04
SCL_min_ (cm)	39 (18–60)	82 (46–108)	33 ± 5 (17–49)	37 (20–52)	64 (50–81)	65 ± 7 (52–88)^g^
Mass (kg)	1.2 (1.0–2.6)	NA	NA	9.0 (1.2–20.7)	NA	44.7 ± 17 (20–105)
BCI	1.8 (1.6–2.0)	NA	NA	1.6 ± 0.01; 1.0–2.0	NA	NA
Life stage	28 J	J, A	69 J	27 J	191 J	35 SA, 5 A
Sex	Unknown	U	U	17 F, 8 M, 2 U	103 F, 48 M, 40 U	30 F, 8 M, 1 U
*N*	28	165–168^h^	69	4–27^h^	190–191^h^	12–39^h^

MCT	Median (range)	Median (range)	Median (range)	Median (range)	Median (range)	Mean ± SD (range) OR median (10–90% quartiles)
Albumin (g/dl)	1.1 (0.7–1.3)	0.9 (0.3–1.8)	1.1 (1.0–1.4)	1.3 (1.0–2.0)	1.1 (0.4–1.7)	1.3 ± 0.3 (0.8–1.6)
A:G ratio	0.42 (0.30–0.50)	0.29 (0.11–0.62)	NA	NA	NA	NA
ALP (U/l)	26 (11–52)	13 (3–76)	67 (51–562)	68 (51–120)	NA	NA
ALT (U/l)	1 (0–28)	1 (0–12)	24 (<10–258)	NA	NA	16 ± 6 (0–29)
AST (U/l)	148 (94–287)	186 (39–951)	194 (<10–844)	79 (13–238)	161 (50–390)	165 (2–255)
BUN (mg/dl)	77.5 (44.0–82.0)	35.5 (2.0–125.0)	101.7 (5.0–188.5)	201.2 (62.1–344.5)	94.4 (33.0–175.9)^i^	82.9 (1.1–107.0)
Calcium (mg/dl)	7.3 (4.7–10.9)	6.9 (2.2–17.1)	8.0 (2.8–12.4)	5.1 (3.1–7.1)	7.6 (5.2–11.6)	7.6 (5.6–8.4)
Ca:P ratio	0.83 (0.28–1.38)	0.88 (0.29–2.03)	NA	NA	0.8 (0.4–1.8)	NA
Chloride (mmol/l)	113 (103–123)	118 (108–127)	NA	116 (100–136)	115 (101–129)	130 ± 11 (107–158)
Cholesterol (mg/dl)	163.5 (92.0–361.0)	155.0 (25.0–494.0)	139.2 (50.3–398.3)	101.0 (60.0–200.0)	NA	75.0 (45.2–200.3)
Creatinine (mg/dl)	0.2 (0.1–0.3)	0.3 (0.1–0.7)	0.4 (0.3–0.8)	NA	NA	0.3 (0.1–0.5)
Globulins (g/dl)	2.6 (1.6–3.4)	3.2 (1.3–5.9)	1.3 (0–2.6)	NA	2.4 (1.3–4.6)	2.9 ± 0.9 (1.0–4.0)
Glucose (mg/dl)	114.0 (88.0–174.0)	95.0 (54.0–171.0)	129.6 (19.8–291.6)	132 (71.0–197.0)	104.4 (45.0–232.2)	106.2 ± 19.8 (70.2–136.8)
Iron (μg/dl)	17.0 (4.0–57.0)	36.0 (7.0–389.0)	NA	NA	NA	NA
LDH (U/l)	61 (19–139)	92 (24–403)	<100 (<100)	NA	NA	572 (6–1376)
PCV (%)	21 (14–32)	NA	28 (17–45)	NA	31 (9–40)	32 ± 5 (18–40)
Phosphorus (mg/dl)	9.3 (5.7–16.8)	8.1 (3.8–15.8)	NA	7.4 (3.3–13.4)	6.8 (3.7–11.2)	6.5 ± 1.2 (4.0–8.1)
Potassium (mmol/l)	3.7 (3.2–4.1)	4.2 (2.2–6.5)	NA	4.5 (3.7–7.3)	4.2 (2.5–6.1)	5.1 ± 2.0 (3.3–13.9)
Sodium (mmol/l)	155 (149–164)	158 (149–179)	NA	150 (136–166)	150 (145–150)	156 ± 11 (135–175)
Total bilirubin (mg/dl)	0.1 (0–0.2)	0.1 (0–1.0)	0.2 (<0.2–0.5)	0.4 (0.2–1.2)	NA	NA
Total protein (g/dl)	3.7 (2.3–4.7)	4.1 (2.0–6.9)	2.4 (2.0–11.0)	3.0 (2.1–4.0)	3.5 (2.1–6.0)	3.7 ± 1.1 (1.6–5.6)
Triglycerides (mg/dl)	139.5 (21.0–637.0)	49.5 (5.0–1859.0)	654.9 (26.6–1858.4)	NA	NA	53.1 (17.7–123.9)
Uric acid (mg/dl)	0.8 (0.5–2.5)	0.8 (0.1–2.3)	1.0 (<0.8–1.7)	1.3 (1.0–2.4)	0.8 (0.1–2.8)	0.7 (0.2–1.2)

Data represent the median or mean ± SD (when given) with the range in parentheses.

Abbreviations: A, adult; A:G, albumin:globulin; Ca:P, calcium:phosphorus; F, female; J, juvenile; M, male; MCT, measure of central tendency; SA, subadult; U, unknown.

^a^Methodology: In-water study; sampling within 30 min of capture.

^b^Data presented in Bolten *et al.* (1994) were further analyzed for this study.

^c^Methodology: Juveniles sampled after a rehabilitation period of 10–195 d.

^d^Methodology: Scoop net capture; placement in tanks overnight; blood draw within 12–18 h after capture.

^e^Methodology: Pound net capture; sampling within 15 min after removal from net; nets were retrieved every 4 d max.

^f^Methodology: Up to 30 min duration trawl captures of juveniles and adults.

^g^Values reported as CCL. They were converted to SCL using Bjorndal *et al.* (2000b).

^h^Range of animals included since the number of data points varied for each test performed.

^i^Values were different between fall and summer; fall values are reported here.

**Figure 1:**
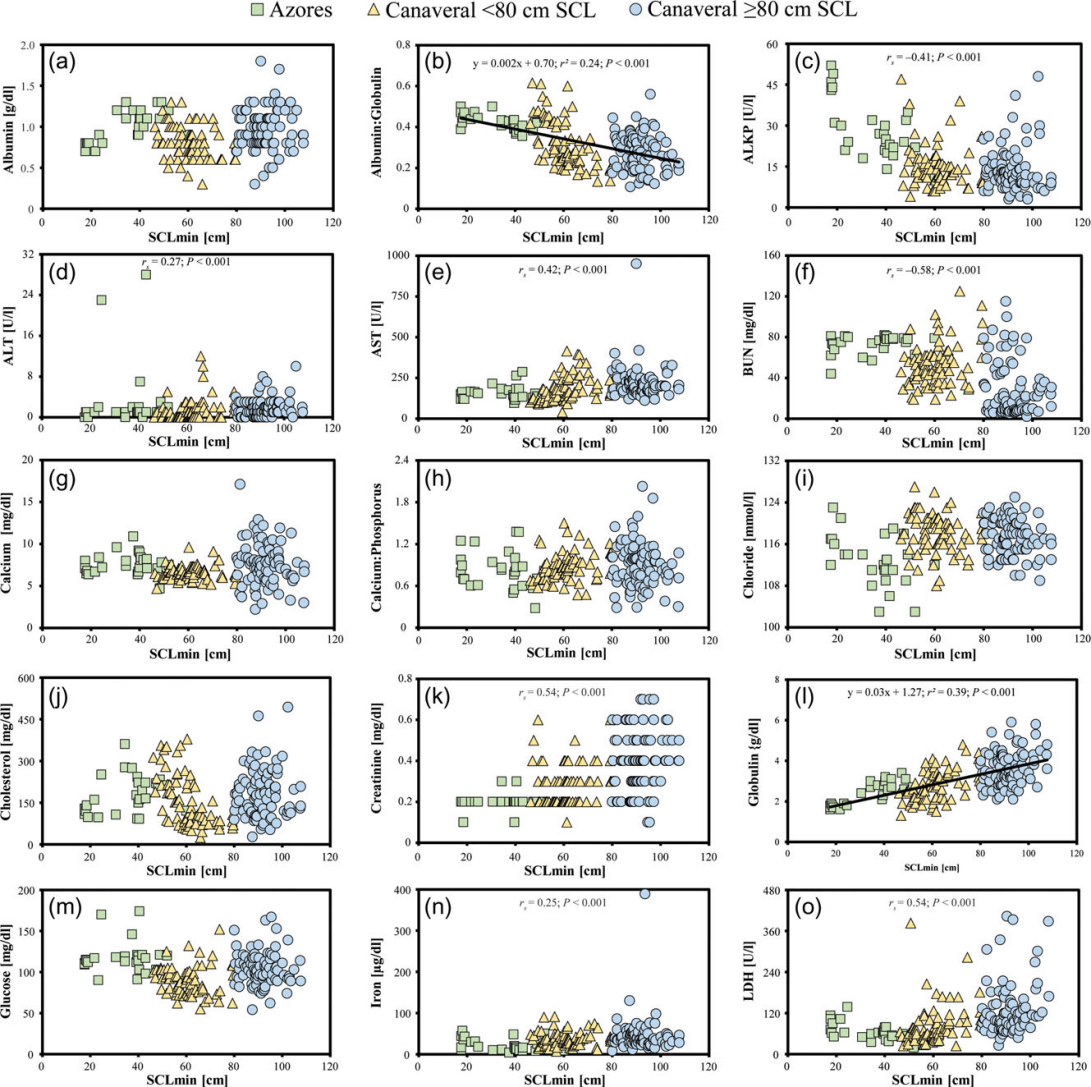

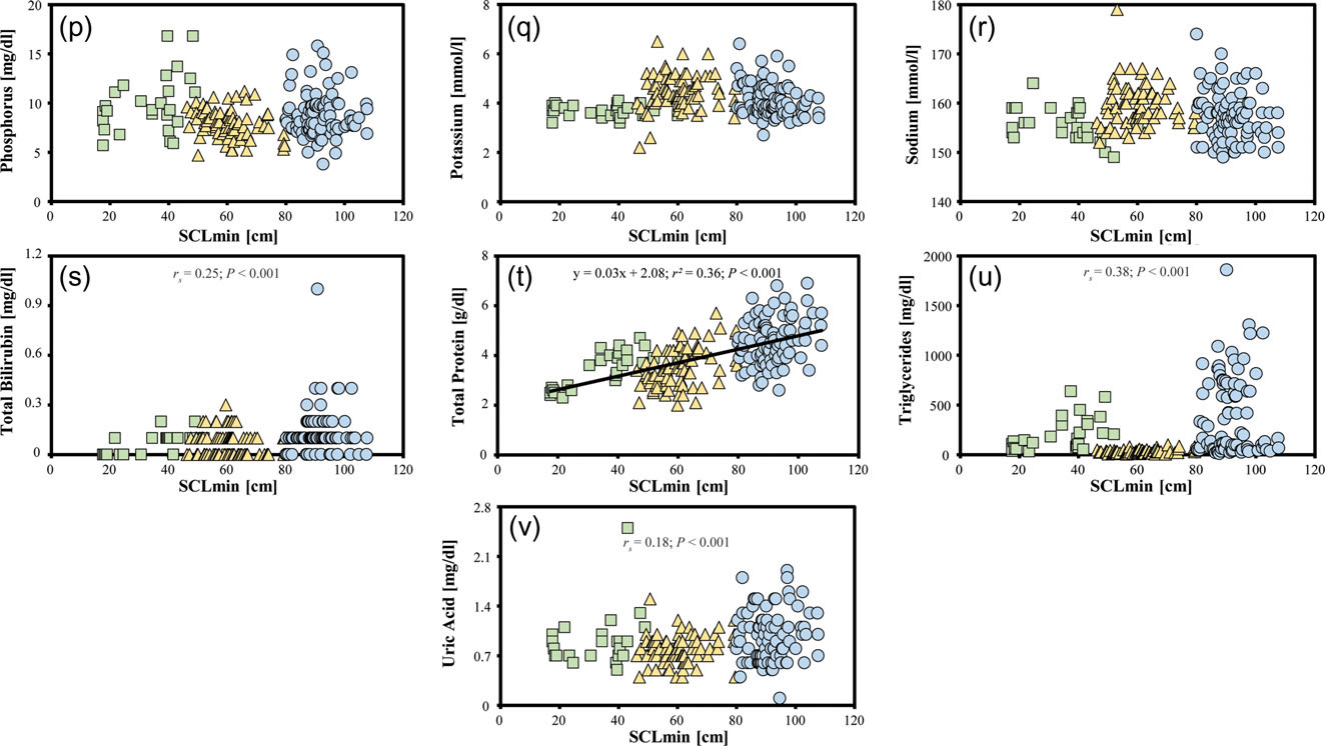
Distribution of blood analytes in relation to size for Azorean loggerheads (green squares), Cape Canaveral loggerheads <80 cm SCL_min_ (yellow triangles), and Cape Canaveral loggerheads ≥80 cm SCL_min_ (blue circles). Results of significant linear regressions (equation included with regression line) or Spearman correlations (*r*_*s*_ value included) for all size classes are included within each panel.

Linear regression or Spearman correlation analysis identified various analytes that significantly increased or decreased with SCL_min_ in Azorean and Cape Canaveral loggerheads as represented in Table 3 and Fig. 1. For Azorean loggerheads, albumin, ALT, globulins, PCV, total bilirubin, total protein and triglycerides significantly increased with increasing SCL_min_, while A:G, ALP, Cl, LDH and Na decreased with increasing SCL_min_. For Cape Canaveral loggerheads <80 cm SCL_min_, ALT, AST, BUN, Ca:P, globulins, LDH, total protein and triglycerides increased with increasing SCL_min_, while albumin, A:G, cholesterol and P decreased with increasing SCL_min_. For Cape Canaveral loggerheads ≥80 cm SCL_min_, globulins, LDH and total protein increased with increasing SCL_min_, while Cl and K decreased with increasing SCL_min_. For all loggerheads combined, ALT, AST, creatinine, globulins, iron, LDH, total bilirubin, total protein, triglycerides and uric acid increased with increasing SCL_min_, while A:G, ALP and BUN decreased with increasing SCL_min._

**Table 3:** Significant linear regressions or Spearman correlations (if the residuals could not be normalized) between SCL_min_ and the measured blood analytes in loggerhead sea turtles from the Azores and Cape Canaveral, Florida USA

Parameter	Azores				Cape Canaveral <80 cm SCL_min_				Cape Canaveral ≥80 cm SCL_min_				Azores and Cape Canaveral combined			
	*r*^2^ or*r*_*s*_	*P*	Direction	*N*	*r*^2^ or*r*_*s*_	*P*	Direction	*N*	*r*^2^ or*r*_*s*_	*P*	Direction	*N*	*r*^2^ or *r*_*s*_	*P*	Direction	*N*
Albumin	**0.47**	**<0.001**	**+**	**28**	**0.09**	**0.01**	**–**	**74**	<0.01	0.60	+	89	0.01	0.12	+	191
Albumin:globulin ratio	**0.40**	**<0.001**	**–**	**28**	**0.37**	**<0.001**	**–**	**74**	0.02	0.17	–	89	**0.24**^a^	**<0.001**	**–**	**191**
ALP	**0.45**	**<0.001**	**–**	**28**	0.12	0.30	–	75	0.18^b^	0.09	–	89	**0.41**^b^	**<0.001**	**–**	**192**
ALT	**0.41**^b^	**0.03**	**+**	**28**	**0.33**^b^	**0.004**	**+**	**75**	0.16^b^	0.15	+	89	**0.27**^b^	**<0.001**	**+**	**192**
AST	0.10^b^	0.60	+	28	**0.16**^a^	**<0.001**	**+**	**75**	0.09^b^	0.40	–	89	**0.42**^b^	**<0.001**	**+**	**192**
BUN	0.31^b^	0.11	+	28	**0.09**^a^	**0.01**	**+**	**75**	0.10^b^	0.38	–	89	**0.58**^b^	**<0.001**	**–**	**192**
Calcium	<0.01	0.92	–	28	0.06^b^	0.60	+	75	0.02	0.17	–	89	0.08^b^	0.29	+	192
Calcium:phosphorus ratio	0.03	0.36	–	28	**0.06**^a^	**0.03**	**+**	**75**	0.01^a^	0.36	–	89	0.10^b^	0.19	+	192
Chloride	**0.23**	**0.01**	**–**	**28**	0.16	0.16	–	75	**0.06**	**0.03**	**–**	**89**	0.08^b^	0.28	+	192
Cholesterol	0.11	0.09	+	28	**0.33**	**<0.001**	**–**	**75**	<0.01^a^	0.86	+	89	0.01^b^	0.93	+	192
Creatinine	0.12^b^	0.53	+	28	0.18^b^	0.13	+	75	0.02	0.18	+	89	**0.54**^b^	**<0.001**	**+**	**192**
Globulins	**0.70**	**<0.001**	**+**	**28**	**0.32**	**<0.001**	**+**	**74**	**0.07**	**0.01**	**+**	**89**	**0.39**	**<0.001**	**+**	**191**
Glucose	0.02^b^	0.91	+	28	0.15^b^	0.20	–	75	<0.01^a^	0.57	–	89	<0.01	0.95	–	192
Iron	0.30^b^	0.12	–	28	0.02^b^	0.87	+	75	0.11^b^	0.32	–	89	**0.25**^b^	**<0.001**	**+**	**192**
LDH	**0.41**	**<0.001**	**–**	**28**	**0.40**^b^	**<0.001**	**+**	**75**	**0.21**^b^	**0.049**	**+**	**89**	**0.54**^b^	**<0.001**	**+**	**192**
PCV	**0.25**	**0.03**	**+**	**20**	**NA**	**NA**	**NA**	**NA**	**NA**	**NA**	**NA**	**NA**	**NA**	**NA**	**NA**	**NA**
Phosphorus	0.06	0.20	+	28	**0.07**	**0.02**	**–**	**75**	<0.01^a^	0.77	–	89	0.03^b^	0.73	–	192
Potassium	0.02	0.49	–	28	0.04^b^	0.74	+	75	**0.26**^b^	**0.02**	**–**	**89**	0.03	0.66	–	192
Sodium	**0.19**	**0.02**	**–**	**28**	0.02^b^	0.85	+	75	0.04	0.08	–	89	0.07	0.31	–	192
Total bilirubin	**0.32**	**0.002**	**+**	**28**	0.10^b^	0.38	–	75	0.08^b^	0.49	+	89	**0.25**^b^	**<0.001**	**+**	**192**
Total protein	**0.67**	**<0.001**	**+**	**28**	**0.24**	**<0.001**	**+**	**75**	**0.07**	**0.01**	**+**	**89**	**0.36**	**<0.001**	**+**	**192**
Triglycerides	**0.14**	**0.047**	**+**	**28**	**0.11**^a^	**0.01**	**+**	**75**	0.03^b^	0.77	+	89	**0.38**^b^	**<0.001**	**+**	**192**
Uric acid	0.09	0.65	+	28	0.13^b^	0.28	+	75	0.01^a^	0.37	+	89	**0.18**^b^	**0.01**	**+**	**192**

Cape Canaveral turtles were divided into two groups: min considered to be juveniles and ≥80 cm considered to be adults. Bold data indicate statistical significance (*p* < 0.05).

^a^Data were square-root transformed to normalize residuals.

^b^Residuals could not be normalized, therefore Spearman correlations were used to determine trends and the Spearman *r*_*s*_ value is shown.

Table 4 presents a comparison of slopes of lines-of-best-fit between Azorean and Cape Canaveral turtles between blood analytes and SCL_min_. We found that the following slopes of the lines-of-best-fit comparing blood analytes to SCL_min_ differed between Azores turtles and Cape Canaveral turtles <80 cm SCL_min_: albumin, A:G, ALP, AST, cholesterol, LDH, P, Na, total bilirubin and triglycerides.

**Table 4:** Comparison of regression line slopes (*b*) between Azorean and Cape Canaveral loggerhead sea turtles <80 cm SCL_min_ using a student’s*t*-test

Parameter	*r*^2^ or *r*_*s*_	Azores *b*	Canaveral *b*	*t*	**df**	*P*
**Albumin**	***r***^**2**^	**0.011**	**–0.008**	**5.081**	**98**	**<0.001**
**Albumin:globulin ratio**	***r***^**2**^	**–0.002**	**–0.009**	**4.411**	**98**	**<0.001**
**ALP**	***r***^**2**^	**–0.577**	**–0.0646**	**–2.600**	**99**	**0.011**
ALT	*r*_*s*_	0.394	0.302	0.438	99	0.662
**AST**	*r*_*s*_	**–0.104**	**0.508**	**–2.791**	**99**	**0.006**
BUN	*r*_*s*_	0.311	0.230	0.373	99	0.710
Calcium	*r*_*s*_	0.040	0.060	–0.087	99	0.931
Calcium: phosphorus ratio	*r*^2^	–0.004	0.003	–0.032	99	0.975
Chloride	***r***^**2**^	–0.193	–0.061	–1.509	99	0.135
**Cholesterol**	***r***^**2**^	**1.863**	**–6.390**	**5.441**	**99**	**<0.001**
Creatinine	*r*_*s*_	0.075	0.164	–0.392	99	0.696
Globulins	***r***^**2**^	0.039	0.055	–1.494	98	0.138
Glucose	*r*_*s*_	0.012	–0.150	0.711	99	0.479
Iron	*r*_*s*_	–0.303	0.020	–1.466	99	0.146
**LDH**	*r*_*s*_	**–0.623**	**0.398**	**–5.454**	**99**	**<0.001**
**Phosphorus**	***r***^**2**^	**0.059**	**–0.048**	**2.153**	**99**	**0.034**
Potassium	*r*_*s*_	–0.146	0.039	–0.814	99	0.418
**Sodium**	***r***_***s***_	**–0.440**	**0.022**	**–2.188**	**99**	**0.031**
**Total bilirubin**	*r*_*s*_	**0.492**	**–0.095**	**2.2927**	**99**	**0.004**
Total protein	***r***^**2**^	0.049	0.048	0.142	98	0.887
**Triglycerides**	***r***_***s***_	**5.227**	**0.075**	**24.274**	**99**	**<0.001**
Uric acid	*r*_*s*_	0.088	0.125	–0.162	99	0.872

The *r*^2^ or *r*_*s*_ column indicates if the slope was calculated from the regression line or the Spearman correlation line. Bold data indicate statistical significance (*p* < 0.05).

## Discussion

This is the first report of blood data from oceanic-juvenile loggerhead turtles of the Azores that documents differences in blood analytes reflective of physiological differences presumptively associated with somatic growth, body size, diet, and environment between oceanic Azorean loggerheads compared to neritic loggerhead turtles in Cape Canaveral. These findings also contribute to the understanding of unique demographic patterns in loggerhead turtles during the oceanic-juvenile stage in the mid-Atlantic Ocean. As described by Bolten *et al.* (1993), the Azorean loggerheads are comparatively smaller than Madeira Archipelago turtles and appear to continue their journey from the Azores to Madeira back to the neritic habitat of the western Atlantic. Delgado *et al.* (2011) present the only available blood chemistry data from loggerheads of the Madeira Archipelago and no other blood chemistry data exist to date regarding oceanic stage juvenile loggerheads. Although analytical methodology differences may have contributed slightly to plasma chemistry data differences, Madeira turtles had comparatively higher albumin, ALP, BUN and K, and lower Ca, P, Na and cholesterol than Azorean turtles. These differences were presumably associated with the substantially different capture and holding technique in Madeira turtles, since the turtles were sampled within 12–18 h post-capture after being held in seawater tanks on land. Since the animals were not fed during the holding time, fasting, osmoregulatory changes (i.e. adjustment to tank water), and possible stress resulted in increased protein breakdown and other metabolic differences reflected in these blood chemistry results and confound a more direct comparison to Azorean plasma chemistry data. Therefore, the following discussion focuses on comparisons between blood data from oceanic Azorean and neritic Cape Canaveral loggerheads that were obtained by identical blood sampling, processing techniques, and analytical methodology, while considering the difference in capture techniques between both locations.

## Packed cell volume: correlation with size

PCV data were only available for Azorean turtles and a comparison with neritic turtles was not possible. As anticipated, the positive size correlation observed in smaller Azorean loggerheads reflected similarly low PCV results when comparing to other studies of immature loggerhead turtles (Deem *et al.*, 2009; Rousselet *et al.*, 2013). This size correlation has been associated with smaller red blood cells and red blood cell volume in smaller turtles (Frair, 1977).

Size-dependent increasing diving activity in larger turtles requires higher oxygen availability through increasing PCV, as previously described in leatherback and loggerhead sea turtles (Stamper *et al.*, 2005; Perrault *et al.*, 2016). In addition, the concurrently observed associations of plasma proteins and somatic growth may have similar application in context of similar trends in PCV in that changes in tissue growth may have various effects on blood volume in growing turtles, although this is primarily well-studied in mammals (Spensley *et al.*, 1987).

## Plasma proteins are notably associated with body size and habitat

The positive size-relevant correlations in albumin, globulins and total protein in Azorean and Cape Canaveral turtles with albumin increasing in smaller oceanic turtles and the A:G ratio decreasing as result from increasing globulins in neritic turtles was expected. These positive correlations can be explained by diet differences and somatic growth, both of which are fundamentally connected. The differences in diet in oceanic and neritic loggerhead turtles have been well documented, and presumably mainly account for the positive correlation with size due to a major diet transition from mainly pelagic and epipelagic coelenterates in oceanic turtles to benthic hard-shelled invertebrates in neritic turtles (Bjorndal, 1997; Bjorndal *et al.*, 2003a; Frick *et al.*, 2009; Jones and Seminoff, 2013). The association of somatic growth and plasma proteins has been well documented in other studies with loggerhead turtles as well as in wild growing green turtles (*Chelonia mydas*) and is presumably associated with changes in metabolism and tissue growth in young animals (Bolten and Bjorndal, 1992: Bolten *et al.*, 1992; Bolten *et al.*, 1994; Osborne *et al.*, 2010; Rousselet *et al.*, 2013; Stacy and Innis, 2017). Azorean loggerheads have the fastest growth rate at smaller body size and the growth rate slows as they increase in size during their oceanic life stage. The growth rate increases again (Bolten, 2003b) following the transition from oceanic to neritic habitats, perhaps due to the dietary shift from this habitat change. The observed positive correlation of total protein with size in oceanic and neritic loggerhead turtles mirrors these growth patterns. The negative correlation of the A:G ratio with increasing size in oceanic and smaller neritic turtles signifies that globulins increase faster than albumin; this is indicated by the slope of the lines-of-best-fit for Azorean loggerheads (albumin slope = 0.011; globulins slope = 0.039; Table 4). An increased immunoglobulin production in neritic turtles may be associated with increased antigen exposure with age, in addition to more frequent antigen exposure in neritic habitats, given the proximity to the shore. Similar associations of immunoglobulins were observed with increasing turtle body size and weight in loggerhead and green sea turtles and in larger compared to smaller adult nesting female leatherback sea turtles (Osborne *et al.*, 2010; Perrault *et al.*, 2014). When comparing to other published studies using similar analytical methodology in loggerhead turtles, the protein data were overall similar (Bolten *et al.*, 1994; Deem *et al.*, 2009; Delgado *et al.*, 2011; Kelly *et al.*, 2015).

A limiting factor with the above considerations is the methodology of albumin quantification by bromocresol green method as used in this study, which inherently results in higher concentrations than compared to the gold standard of albumin measurement by protein electrophoresis based on total protein quantification by Biuret method (Macrelli *et al.*, 2013). However, comparison of data from both populations that is based on the use of the same chemistry analyzer is acceptable, considering the limitation with this methodology. When removing albumin from the above conclusions, the correlations of total protein are applicable and underline the biological relevance of the discussed findings.

## Tissue enzyme activities indicate differences in somatic growth

As observed in this study, tissue enzyme activities in sea turtles in general are quite variable and increases in plasma activities may be difficult to interpret (Anderson *et al.*, 2013; Stacy and Innis, 2017). However, the overt positive size-correlations of ALT, a tissue enzyme with inherently low tissue specificity and high variability (Anderson *et al.*, 2013), in oceanic Azorean and neritic Cape Canaveral turtles <80 cm SCL_min_ suggest faster tissue growth at smaller turtle size, given that this correlation was absent in larger Cape Canaveral turtles. This finding goes along with the negative correlation of ALP with size and a faster decrease in Azorean turtles, given a probable association with bone growth, including faster growth, at smaller turtle size. These observations match the conclusions regarding size- and growth pattern-relevant correlations with total protein above.

AST without data for CK is difficult to fully interpret, since AST has high activities in muscle as well as in other tissues, while high CK activities are mainly associated with leakage from muscle tissue (Stacy and Innis, 2017). Therefore, increased AST with concurrently low CK reduces the probability of AST originating from muscle tissue. Since CK measurement was not included in this study, the concurrently higher AST and LDH activities in larger Cape Canaveral turtles may suggest some degree of muscle damage possibly from capture in trawlers or differences in activity in larger turtles, although other metabolic differences resulting from diet and/or habitat may have contributed. Comparisons to other studies in enzyme activities are of limited value, given the lack of CK data and differences in capture techniques.

## Electrolytes and minerals suggest population differences

Na and Cl follow interesting trends in Azorean turtles with a negative size-correlation that is also reflected in selectively lower Na in oceanic-juvenile in comparison to larger neritic turtles. Plasma P was negatively correlated with size in smaller neritic (<80 cm SCL_min_) Cape Canaveral turtles in addition to a negative size-correlation of K in larger neritic Cape Canaveral turtles (≥80 cm SCL_min_). These observations appear to be driven by differences in diet, habitat, or possibly osmoregulation unique to both populations. Diet differences are well documented as discussed above and a difference in dietary Na content may contribute to this observation; however, considering the differences in developmental stage, osmoregulation in smaller turtles may be associated with somatic growth (i.e. developing or growing salt gland) or a different osmoregulatory response in smaller turtles in oceanic habitats with different salinity compared to neritic habitats. The negative size-correlations of P and K in Cape Canaveral turtles point towards changes in bone metabolism and/or diet (Stacy and Innis, 2017).

## Lipids and other plasma analytes (e.g. glucose, iron, BUN, bilirubin) reflect diet and size-relevant correlations

Plasma lipid correlations also reflect the above-mentioned diet transition between oceanic and neritic populations. Cholesterol and triglyceride concentrations were comparatively lower in Azorean compared to Cape Canaveral turtles, and triglycerides increased much faster in Azorean turtles (Azorean cholesterol slope = 1.863; Canaveral cholesterol slope = –6.390; Azorean triglycerides slope = 5.227; Canaveral triglycerides slope = 0.075; Table 4), with a concurrent positive size-correlation of triglycerides in Azorean loggerheads and smaller Cape Canaveral loggerheads.

Glucose appears to be similar when comparing oceanic and neritic loggerhead turtles and ranges are similar to other studies. This is a closely regulated plasma analyte that can be erroneously increased in non-fasted samples, and can change quickly in association with physiological responses or various diseases (Stacy and Innis, 2017). For example, juvenile loggerheads after rehabilitation reportedly have comparatively higher plasma glucose, which may be confounded by non-fasting samples, captive diet and/or stress from handling (Casal *et al.*, 2009). Plasma glucose can increase rapidly as part of a stress response in most animals, including sea turtles. Increased plasma glucose was associated with increased plasma corticosterone and entanglement time in gillnet-captured Kemp’s ridley (*Lepidochelys kempii*) sea and green turtles (Snoddy *et al.*, 2009). Tangle net-captured loggerhead sea turtles had lower corticosterone concentrations than those captured by trawl, with a rapid increase of corticosterone within the first hour after capture with both capture techniques (Gregory *et al.*, 1996). Despite this anticipation of possibly higher plasma glucose in trawler-captured Cape Canaveral turtles, data were within similar ranges for Azorean turtles and similar to reported data in various health assessment studies (Stacy and Innis, 2017). Based on these findings and considering the short mean average trawl duration of 25.5 min, we conclude that the capture techniques used in this study may have resulted in a low-level stress response that was not significant enough to cause any overt glucose derangements in captured turtles from both locations. Analytes other than glucose that may be associated with a physiologic stress response were not measured in this study.

Plasma iron was also within stable ranges within populations, which is expected in healthy turtles with concurrent stable plasma protein concentrations, since the analytical method of plasma iron quantifies the protein-bound fraction of plasma iron (Stacy and Innis, 2017).

BUN of oceanic and neritic loggerheads was within expected ranges for the species, with an interesting positive size correlation of smaller Cape Canaveral turtles, possibly associated with the previously discussed dietary shift in this life stage during the transition from oceanic to neritic waters. Creatinine is generally low and not considered diagnostically valuable in sea turtles (Stacy and Innis, 2017). Although no size-relevant correlations were observed, larger Cape Canaveral turtles appear to trend higher which may be associated with larger muscle mass and body size.

The limited diagnostic value of bilirubin in reptiles prevents interpretation of the finding of a positive size-correlation in smaller Cape Canaveral turtles. Although hemolysis was absent in the samples used for analysis in this study, lipids and BUN may interfere with the analytical methodology and may have resulted in the variability of results, considering that the samples in this study were not fasted; however, the objective of this study was to provide a ‘snap shot’ of blood data in healthy Azorean loggerhead turtles that were sampled very quickly after capture without interference from holding, stress or fasting. The above-mentioned sample interference is important to consider given the lack of biliverdin reductase in reptiles and thus generally very low bilirubin concentrations that are often either undetectable or not reported in many studies reporting blood data (Campbell, 2012; Rousselet *et al.*, 2013; Stacy and Innis, 2017).

## Conclusions

This report provides blood data from oceanic-juvenile loggerhead turtles from the Azores representative of blood data from turtles caught during an in-water study, i.e. the data presented herein were unlikely affected by significant capture effects and are representative of a true ‘snap shot’ of foraging turtles at this life stage in this unique geographic location. Because of similar methodology, it was also possible to compare these data from Azorean loggerhead turtles to those from the Western Atlantic Ocean and to identify major physiological differences related to size, somatic growth, diet and environment, confirming biologically relevant changes during life stage development and migration. Reference intervals for hematology and plasma chemistry data provide essential baseline information for the evaluation of an individual turtle (e.g. stranding and rehabilitation) or are applicable to answering questions on a population level (e.g. cold-stunning) (Stacy and Innis, 2017). The data presented herein will be useful for the development of conservation strategies and for future temporal and spatial investigations of the Azorean loggerhead sea turtle population.

## Supplementary Material

Supplementary Data

Supplementary Data

## References

[coy006C10000] Anderson ET, Socha VL, Gardner J, Byrd L, Manire CA (2013) Tissue enzyme activities in the loggerhead sea turtle (Caretta caretta). J Zoo Wildl Med 44(1): 62–69. 23505704 10.1638/1042-7260-44.1.62

[coy006C1] Avens L, Goshe LR, Pajuelo M, Bjorndal KA, MacDonald BD, Lemons GE, Bolten AB, Seminoff JA (2013) Complementary skeletochronology and stable isotope analyses offer new insight into juvenile loggerhead sea turtle (*Caretta caretta*) oceanic stage duration and growth dynamics. Mar Ecol Prog Ser 491: 235–251.

[coy006C2] Bjorndal K (1997) Foraging ecology and nutrition of sea turtles In Lutz PL, Musick JA, eds The Biology of Sea Turtles, Vol I CRC Press, Boca Raton, pp 199–231.

[coy006C3] Bjorndal KA, Bolten AB, Dellinger T, Delgado C, Martins HR (2003a) Compensatory growth in oceanic loggerhead sea turtles: response to a stochastic environment. Ecology 84: 1237–1249.

[coy006C4] Bjorndal KA, Bolton AB, Chaloupka MY (2000a) Green turtle somatic growth model: evidence for density dependence. Ecol Appl 10: 269–282.

[coy006C5] Bjorndal KA, Bolten AB, Martins HR (2000b) Somatic growth model of juvenile loggerhead sea turtles *Caretta caretta*: duration of pelagic stage. Mar Ecol Prog Ser 202: 265–272.

[coy006C6] Bjorndal KA, Bolten AB, Martins HR (2003b) Estimates of survival probabilities for oceanic-stage loggerhead sea turtles (*Caretta caretta*) in the North Atlantic. Fish Bull 101: 732–736.

[coy006C7] Bolten AB (2003a) Active swimmers—passive drifters: the oceanic juvenile stage of loggerheads in the Atlantic system In Bolten AB, Witherington BE, eds Loggerhead Sea Turtles. Smithsonian Institution Press, Washington, DC, pp 63–78.

[coy006C8] Bolten AB (2003b) Variation in sea turtle life history patterns: neritic vs. oceanic developmental stages In Lutz PL, Musick JA, Wyneken J, eds The Biology of Sea Turtles Volume II. CRC Press, Boca Raton, FL, pp 243–258.

[coy006C9] Bolten AB, Bjorndal KA (1992) Blood profiles for a wild population of green turtles (*Chelonia mydas*) in the southern Bahamas: size-specific and sex-specific relationships. J Wildl Dis 28: 407–413. 1512872 10.7589/0090-3558-28.3.407

[coy006C10] Bolten AB, Bjorndal KA, Eliazar PJ, Gregory LF (1994) Seasonal abundance, size distribution, and blood biochemical values of loggerheads (*Caretta caretta*) in Port Canaveral Ship Channel, Florida. NOAA Technical Memorandum NMFS-SEFSC-353.

[coy006C11] Bolten AB, Bjorndal KA, Martins HR, Dellinger T, Biscoito MJ, Encalada SE, Bowen BW (1998) Transatlantic developmental migrations of loggerhead sea turtles demonstrated by mtDNA sequence analysis. Ecol Appl 8: 1–7.

[coy006C12] Bolten AB, Jacobson ER, Bjorndal KA (1992) Effects of anticoagulant and autoanalyzer on blood biochemical values of loggerhead sea turtles (*Caretta caretta*). Am J Vet Res 53: 2224–2227. 1476302

[coy006C13] Bolten AB, Martins HR, Bjorndal KA, Gordon J (1993) Size distribution of pelagic-stage loggerhead sea turtles (*Caretta caretta*) in the waters around the Azores and Madeira. Arquipelago 11A: 49–54.

[coy006C14] Campbell TW (2012) Clinical pathology of reptiles In Thrall MA, Weiser G, Allison R, Campbell T, eds Veterinary Hematology and Clinical Chemistry, Ed 2 John Wiley & Sons Inc, Ames, pp 601–604.

[coy006C15] Casal AB, Camacho M, López‐Jurado LF, Juste C, Orós J (2009) Comparative study of hematologic and plasma biochemical variables in Eastern Atlantic juvenile and adult nesting loggerhead sea turtles (*Caretta caretta*). Vet Clin Pathol 38: 213–218. 19192261 10.1111/j.1939-165X.2008.00106.x

[coy006C16] Deem SL, Norton TM, Mitchell M, Segars A, Alleman AR, Cray C, Poppenga RH, Dodd M, Karesh WB (2009) Comparison of blood values in foraging, nesting, and stranded loggerhead turtles (*Caretta caretta*) along the coast of Georgia, USA. J Wildl Dis 45: 41–56. 19204334 10.7589/0090-3558-45.1.41

[coy006C17] Delgado C, Valente A, Quaresma I, Costa M, Dellinger T (2011) Blood biochemistry reference values for wild juvenile loggerhead sea turtles (*Caretta caretta*) from Madeira archipelago. J Wildl Dis 47: 523–529. 21719817 10.7589/0090-3558-47.3.523

[coy006C18] Florida Fish and Wildlife Conservation Commission (FFWCC) 2016 FFWCC Marine Turtle Conservation Handbook. Rule 68E-1.004, 2016. Available online: myfwc.com/media/4112794/fwc-mtconservationhandbook.pdf. Accessed November 5 2017.

[coy006C19] Flint M, Morton JM, Limpus CJ, Patterson-Kane JC, Mills PC (2010) Reference intervals for plasma biochemical and hematologic measures in loggerhead sea turtles (*Caretta caretta*) from Moreton Bay, Australia. J Wildl Dis 46: 731–741. 20688679 10.7589/0090-3558-46.3.731

[coy006C20] Frair W (1977) Sea turtle red blood cell parameters correlated with carapace lengths. Comp Biochem Physiol 56: 467–472.

[coy006C21] Frick MG, Williams KL, Bolten AB, Bjorndal KA, Martins HR (2009) Foraging ecology of oceanic-stage loggerhead turtles (*Caretta caretta*). Endang Species Res 9: 91–97.

[coy006C22] Friedrichs KR, Harr KE, Freeman KP, Szladovits B, Walton RM, Barnhart KF, Blanco‐Chavez J (2012) ASVCP reference interval guidelines: determination of de novo reference intervals in veterinary species and other related topics. Vet Clin Pathol 41: 441–453. 23240820 10.1111/vcp.12006

[coy006C23] Gregory LF, Gross TS, Bolten AB, Bjorndal KA, Guillette LG Jr (1996) Plasma corticosterone concentrations associated with acute captivity stress in wild loggerhead sea turtles (*Caretta caretta*). Gen Comp Endocrinol 104: 312–320. 8954764 10.1006/gcen.1996.0176

[coy006C24] Jones TT, Seminoff JA (2013) Feeding biology: advances from field-based observations, physiological studies, and molecular techniques In Wyneken J, Lohmann KJ, Musick JA, eds The Biology of Sea Turtles, Vol III CRC Press, Boca Raton, pp 211–247.

[coy006C25] Kelly TR, McNeill JB, Avens L, Hall AG, Goshe LR, Hohn AA, Godfrey MH, Mihnovets AN, Cluse WM, Harms CA (2015) Clinical pathology reference intervals for an in-water population of juvenile loggerhead sea turtles (*Caretta caretta*) in Core Sound, North Carolina, USA. PLoS One 10: e0115739 10.1371/journal.pone.0115739. 25738772 PMC4349656

[coy006C26] Macrelli R, Ceccarelli MM, Fiorucci L (2013) Determination of serum albumin concentration in healthy and diseased Hermann’s tortoises (*Testudo hermanni*): a comparison using electrophoresis and the bromocresol green dye-binding method. J Herp Med Surg 23: 20–24.

[coy006C27] NMFS and USFWS (2011) Endangered and threatened species. Determination of nine distinct population segments of loggerhead sea turtles as endangered or threatened. Fed Regist 76(184): 58868–58952.

[coy006C28] Osborne AG, Jacobson ER, Bresette MJ, Singewald D, Scarpino R, Bolten AB (2010) Reference intervals and relationships between health status, carapace length, body mass, and water temperature, and concentrations of plasma protein and protein fractions of the Atlantic loggerhead sea turtle (*Caretta caretta*) and the green turtle (*Chelonia mydas*). J Am Vet Med Assoc 237: 561–567. 20807135 10.2460/javma.237.5.561

[coy006C29] Perrault JR, Page-Karijan A, Miller DL (2016) Nesting leatherback sea turtles (*Dermochelys coriacea*) packed cell volumes indicate decreased foraging during reproduction. Mar Biol 163: 232.

[coy006C30] Perrault JR, Wyneken J, Page-Karjian A, Merrill A, Miller DL (2014) Seasonal trends in nesting leatherback turtle (*Dermochelys coriacea*) serum proteins further verify capital breeding hypothesis. Conserv Physiol 2, 10.1093/conphys/cou002. PMC4732470 27293623

[coy006C31] Rousselet E, Stacy NNI, LaVictoire K, Higgins BM, Tocidlowski ME, Flanagan JP, Godard-Codding CA (2013) Hematology and plasma biochemistry analytes in five age groups of immature, captive-reared loggerhead sea turtles (*Caretta caretta*). J Zoo Wildl Med 44: 859–874. 24450044 10.1638/2012-0162R1.1

[coy006C32] Snoddy JE, Landon M, Blanvillain G, Souhwood A (2009) Blood biochemistry of sea turtles captures in gillnets in the lower cape fear river, North Carolina, USA. J Wildl Manag 73: 1394–1401.

[coy006C33] Spensley MS, Carlson GP, Harrold D (1987) Plasma, red blood cell, total blood, and extracellular fluid volumes in healthy horse foals during growth. Am J Vet Res 48: 1703–1707. 3434917

[coy006C34] Stacy NI, Innis CJ (2017) Clinical pathology In Manire CA, Norton TM, Stacy BA, Harms CA, Innis CJ, eds Sea Turtle Health and Rehabilitation. J. Ross Publishing, Plantation, FL, pp 147–207.

[coy006C35] Stamper MA, Harms C, Epperly SP, Braun-McNeill J, Avens L, Stoskopf MK (2005) Relationship between barnacle epibiotic load and hematologic parameters in loggerhead sea turtles (*Caretta caretta*), a comparison between migratory and residential animals in Pamlico Sound, North Carolina. J Zoo Wildl Med 36: 635–641. 17312720 10.1638/04-074.1

