# Supplementary material for: Blood analytes of oceanic-juvenile loggerhead sea turtles (Caretta caretta) from Azorean waters: reference intervals, size-relevant correlations and comparisons to neritic loggerheads from western Atlantic coastal waters
Source: Conserv Physiol. 2018 Feb 16;6(1):coy006. doi: 10.1093/conphys/coy006 (PMC5814815; doi:10.1093/conphys/coy006)
Supplement: Supplementary Data [file coy006supplementaltable1_morphometricsandbiochemistrywithreferenceintervalssiunits.docx]

| **Supplemental Table 1**. Morphometrics, body condition index, and blood analyte data for oceanic-juvenile loggerhead sea turtles from the Azores sampled in November 1990 in SI units. Reference intervals represent the 90% confidence interval. | | | | | | |  |
| --- | --- | --- | --- | --- | --- | --- | --- |
| Parameter | Mean±SD | Median | Range | *N* | Lower limit (90% CI) | Upper limit (90% CI) | |
| SCL_min_ (cm) | 34.9±12.1 | 39.4 | 17.6–60.0 | 28 | NA | NA | |
| Mass (kg) | 1.5±0.6 | 1.2 | 1.0–2.6 | 8 | NA | NA | |
| BCI | 1.8±0.1 | 1.8 | 1.6–2.0 | 8 | NA | NA | |
| Albumin (g/l) | 10.1±1.9 | 10.5 | 7.0–13.0 | 28 | 7.0 (5.9–8.0) | 13.2 (12.1–14.2) | |
| Albumin:globulin ratio | 0.42±0.04 | 0.42 | 0.30–0.50 | 28 | 0.35 (0.32–0.37) | 0.49 (0.47–0.51) | |
| ALKP (µkat/l) | 0.48±0.17 | 0.43 | 0.18–0.87 | 28 | 0.19 (0.09–0.28) | 0.76 (0.67–0.86) | |
| ALT (µkat/l ) | 0.05±0.11 | 0.02 | 0–0.47 | 28 | 0 (0)^a^ | 0.22 (0.09–0.50)^a^ | |
| AST (µkat/l) | 2.58±0.74 | 2.46 | 1.57–4.79 | 28 | 1.70 (1.53–1.89)^a^ | 3.98 (3.31–4.92)^a^ | |
| BUN (mmol/l) | 26.3±3.2 | 27.7 | 15.7^b^–29.3 | 28 | 21.0 (0–24.1)^a^ | 29.5 (28.8–30.1)^a^ | |
| Calcium (mmol/l) | 1.9±0.3 | 1.9 | 1.2–2.7 | 28 | 1.4 (1.2–1.6) | 2.4 (2.2–2.6) | |
| Calcium:phosphorus | 0.65±0.20 | 0.63 | 0.22–1.07 | 28 | 0.31 (0.20–0.42) | 0.98 (0.87–1.09) | |
| Chloride (mmol/l) | 113.1±4.9 | 113.0 | 103.0–123.0 | 28 | 105.1 (102.4–107.7) | 121.1 (118.4–123.7) | |
| Cholesterol (mmol/l) | 4.6±1.8 | 4.3 | 2.4–9.3 | 28 | 1.7 (0.7–2.7) | 7.5 (6.2–8.5) | |
| Creatinine (µmol/l) | 17.7±3.4 | 17.7 | 8.8–26.5 | 28 | 12.1 (10.2–14.0)^c^ | 23.3 (21.4–25.2)^c^ | |
| Globulin (g/l) | 24.4±5.6 | 26.0 | 16.0–34.0 | 28 | 14.4 (12.2–18.0)^d^ | 34.6 (32.0–37.0)^d^ | |
| Glucose (mmol/l) | 6.4±1.1 | 6.3 | 4.9–9.7 | 28 | 5.1 (4.9–5.4)^a^ | 8.4 (7.5–9.7)^a^ | |
| Iron (µmol/l) | 4.0±2.7 | 3.0 | 0.7–10.2 | 28 | 0 (0–1.1) | 8.4 (6.9–9.9) | |
| LDH (µkat/l ) | 1.07±0.44 | 1.01 | 0.32–2.30 | 28 | 0.47 (0.35–0.62)^a^ | 1.89 (1.55–2.27)^a^ | |
| PCV (%) | 22.0±5.0 | 21.0 | 14.0–32.0 | 20 | 14.0 (11.0–17.0) | 30.0 (27.0–33.0) | |
| Phosphorus (mmol/l) | 3.2±0.9 | 3.0 | 1.8–5.4 | 28 | 1.6 (1.1–2.1) | 4.7 (4.2–5.2) | |
| Potassium (mmol/l) | 3.7±0.2 | 3.7 | 3.2–4.1 | 28 | 3.3 (3.2–3.4) | 4.1 (3.9–4.2) | |
| Sodium (mmol/l) | 155.7±3.3 | 155.0 | 149.0–164.0 | 28 | 150.3 (148.5–152.1) | 161.1 (159.3–162.8) | |
| Total bilirubin (µmol/l) | 1.15±1.04 | 1.7 | 0–3.42 | 28 | 0 (0-0.01) | 2.86 (2.30–3.43) | |
| Total protein (g/l) | 34.5±7.3 | 36.5 | 23.0–47.0 | 28 | 21.4 (18.5–26.9)^d^ | 47.9 (44.5–51.4)^d^ | |
| Triglycerides (mmol/l) | 2.24±1.89 | 1.58 | 0.24–7.20 | 28 | 0.33 (0.18–0.57)^a^ | 6.52 (4.18–10.00)^a^ | |
| Uric acid (µmol/l) | 53.3±21.7 | 47.6 | 29.7–148.7 | 28 | 34.2 (31.3–37.8)^a^ | 86.3 (68.1–119.4)^a^ | |
| ^a^ ALT, AST, BUN, glucose, LDH, triglycerides, and uric acid reference intervals calculated using Box Cox transformations, as data were non-normal  ^b^ 15.7 mmol/l is an outlier; the next lowest value is 20.3 mmol/l  ^c^ Creatinine could not be transformed to a normal distribution and robust methods of determining reference intervals could not be utilized  ^d^ Globulin and total protein reference intervals are reported using the robust method | | | | | | | |
