# Supplementary material for: Blood analytes of oceanic-juvenile loggerhead sea turtles (Caretta caretta) from Azorean waters: reference intervals, size-relevant correlations and comparisons to neritic loggerheads from western Atlantic coastal waters
Source: Conserv Physiol. 2018 Feb 16;6(1):coy006. doi: 10.1093/conphys/coy006 (PMC5814815; doi:10.1093/conphys/coy006)
Supplement: Supplementary Data [file coy006supplementaltable2_comparisonstable.docx]

| **Supplemental Table 2**. Comparison of published blood analytes in loggerhead sea turtles in standard international (SI) units. Data represent the median or mean±SD (when given) with the range in parentheses. | | | | | | |
| --- | --- | --- | --- | --- | --- | --- |
| Reference | This study: Azores^a^ | This study: Cape Canaveral^a,b^ | Casal *et al.* (2009)^c^ | Delgado *et al.* (2011)^d^ | Kelly *et al.* (2015)^e^ | Deem *et al.* (2009)^f^ |
| Location | Azores; oceanic | Florida USA; neritic | Cape Verde; rehab | Madeira; oceanic | North Carolina USA; neritic | Florida/Georgia USA; neritic |
| Date | Nov 90 | Mar 92–Feb 93 | Aug–Sep 04 | May–Jul 06 | May–Nov 04–07 | May–Sep 00–04 |
| SCL_min_ (cm) | 39 (18–60) | 82 (46–108) | 33±5 (17–49) | 37 (20–52) | 64 (50–81) | 65±7 (52–88)^g^ |
| Mass (kg) | 1.2 (1.0–2.6) | NA | NA | 9.0 (1.2–20.7) | NA | 44.7±17 (20–105) |
| BCI | 1.8 (1.6–2.0) | NA | NA | 1.6±0.01; 1.0–2.0 | NA | NA |
| Life stage | 28 J | J, A | 69 J | 27 J | 191 J | 35 SA, 5 A |
| Sex | Unknown | U | U | 17 F, 8 M, 2 U | 103 F, 48 M, 40 U | 30 F, 8 M, 1 U |
| *N* | 28 | 165–168^h^ | 69 | 4–27^h^ | 190–191^h^ | 12–39^h^ |
| MCT | Median (range) | Median (range) | Median (range) | Median (range) | Median (range) | Mean±SD (range) OR  median (10–90% quartiles) |
| Albumin (g/l) | 10.5 (7.0–13.0) | 9.0 (3.0–18.0) | 11.0 (10.0–14.0) | 13.0 (10.0–20.0) | 11.0 (4.0–17.0) | 13.0±3.0 (8.0–16.0) |
| A:G ratio | 0.42 (0.30–0.50) | 0.29 (0.11–0.62) | NA | NA | NA | NA |
| ALKP (µkat/l) | 0.43 (0.18–0.87) | 0.21 (0.05–1.27) | 1.12 (0.85–9.37) | 1.13 (0.85–2.00) | NA | NA |
| ALT (µkat/l) | 0.02 (0–0.47) | 0.02 (0–0.20) | 0.41 (<0.17–4.39) | NA | NA | 0.27±0.10 (0–0.49) |
| AST (µkat/l) | 2.46 (1.57–4.79) | 3.09 (0.65–15.79) | 3.22 (<0.17–14.01) | 1.30 (0.22–3.95) | 2.67 (0.83–6.47) | 2.74 (0.03–4.23) |
| BUN (mmol/l) | 27.7 (15.7–29.3) | 12.7 (0.7–44.6) | 36.3 (1.8–67.3) | 71.8 (22.2–123.0) | 33.7 (11.8–62.8)^i^ | 29.6 (0.4–38.2) |
| Calcium (mmol/l) | 1.9 (1.2–2.7) | 1.7 (0.6–4.3) | 2.0 (0.7–3.1) | 1.3 (0.8–1.8) | 1.9 (1.3–2.9) | 1.8 (1.4–2.1) |
| Ca:P ratio | 0.65 (0.22–1.07) | 0.68 (0.22–1.57) | NA | NA | 1.03 (0.51–2.31) | NA |
| Chloride (mmol/l) | 113.0 (103.0–123.0) | 118.0 (108.0–127.0) | NA | 116.0 (100.0–136.0) | 115.0 (101.0–129.0) | 130±11 (107–158) |
| Cholesterol (mmol/l) | 4.3 (2.4–9.3) | 4.0 (0.7–12.8) | 3.6 (1.3–10.3) | 2.6 (1.6–5.2) | NA | 1.9 (1.2–4.5) |
| Creatinine (µmol/l) | 17.7 (8.8–26.5) | 26.5 (8.8–61.9) | 31.8 (<26.5–70.7) | NA | NA | 26.5 (8.8–44.2) |
| Globulin (g/l) | 26.0 (16.0–34.0) | 32.0 (13.0–59.0) | 13.0 (0–26.0) | NA | 24.0 (13.0–46.0) | 29.0±9.0 (10.0–40.0) |
| Glucose (mmol/l) | 6.3 (4.9–9.7) | 5.3 (3.0–9.5) | 7.2 (1.1–16.2) | 7.3 (3.9–10.9) | 5.8 (2.5–12.9) | 5.9±1.1 (3.9–7.6) |
| Iron (µmol/l) | 3.0 (0.7–10.2) | 6.4 (1.3–69.6) | NA | NA | NA | NA |
| LDH (µkat/l) | 1.01 (0.32–2.30) | 1.53 (0.4–6.7) | <1.67 (<1.67) | NA | NA | 9.54 (0.10–22.94) |
| PCV (%) | 21.0 (14.0–32.0) | NA | 28 (17–45) | NA | 31 (9–40) | 32±5 (18–40) |
| Phosphorus (mmol/l) | 3.0 (1.8–5.4) | 2.6 (1.2–5.1) | NA | 2.4 (1.1–4.3) | 2.2 (1.2–3.6) | 2.1±0.4 (1.3–2.6) |
| Potassium (mmol/l) | 3.7 (3.2–4.1) | 4.2 (2.2–6.5) | NA | 4.5 (3.7–7.3) | 4.2 (2.5–6.1) | 5.1±2.0 (3.3–13.9) |
| Sodium (mmol/l) | 155.0 (149.0–164.0) | 158.0 (149.0–179.0) | NA | 149.8 (135.9–166.2) | 156.0 (145.0–168.0) | 156.0±11.0 (135.0–175.0) |
| Total bilirubin (µmol/l) | 1.7 (0–3.42) | 1.7 (0–17.1) | 3.4 (<3.4–8.5) | 5.7 (3.4–20.5) | NA | NA |
| Total protein (g/l) | 36.5 (23.0–47.0) | 41.0 (20.0–69.0) | 24.0 (20.0–110.0) | 30.0 (21.0–40.0) | 35.0 (21.0–60.0) | 37.0±11.0 (16.0–56.0) |
| Triglycerides (mmol/l) | 1.6 (0.2–7.2) | 0.6 (0.1–21.0) | 7.4 (0.3–21.0) | NA | NA | 0.6 (0.2–1.4) |
| Uric acid (µmol/l) | 47.6 (29.7–148.7) | 47.6 (6.0–136.8) | 60.0 (50.0–100.0) | 77.3 (59.5–142.8) | 47.6 (5.9–166.5) | 41.6 (11.9–71.4) |
| Abbreviations: A, adult; A:G, albumin:globulin; Ca:P, calcium:phosphorus; F, female; J, juvenile; M, male; MCT, measure of central tendency; SA, subadult; U, unknown  ^a^ Methodology: In-water study; sampling within 30 min of capture  ^b^ Data presented in Bolten et al. (1994) were further analyzed for this study  ^\c^ Methodology: Juveniles sampled after a rehabilitation period of 10–195 d  ^d^ Methodology: Scoop net capture; placement in tanks overnight; blood draw within 12–18 hrs after capture  ^e^ Methodology: Pound net capture; sampling within 15 min after removal from net; nets were retrieved every 4 d max  ^f^ Methodology: Up to 30 min duration trawl captures of juveniles and adults  ^g^ Values reported as CCL. They were converted to SCL using Bjorndal *et al.* (2000)  ^h^ Range of animals included since the number of data points varied for each test performed  ^i^ Values were different between fall and summer; fall values are reported here | | | | | | |
